# Clinical, laboratory, and radiological features influencing admission DWI-ASPECTS in stroke patients with middle cerebral artery occlusion undergoing mechanical thrombectomy

**DOI:** 10.1007/s10072-026-08903-x

**Published:** 2026-03-07

**Authors:** Carmelo Tiberio Currò, Antonio Ciacciarelli, Giulia Fiume, Davide Vicari, Anna Gardin, Concetto Fabio Vecchio, Alessandra Coglitore, Mirta Longo, Giuseppe Trimarchi, Cristina Dell’Aera, Masina Cotroneo, Rosa Fortunata Musolino, Antonio Toscano, Sergio Lucio Vinci, Enricomaria Mormina, Paolino La Spina, Agostino Tessitore

**Affiliations:** 1 Department of Brain and Behavioural Sciences, University of Pavia, Pavia, Italy; 2 IRCCS Fondazione C. Mondino, Via Mondino, 2, Pavia, 27100 Italy; 3 Emergency Department, Stroke Unit, Sapienza University of Rome, Rome, Italy; 4 IRCCS Centro Neurolesi Bonino-Pulejo, Messina, Italy; 5 Department of Clinical and Experimental Medicine, University of Messina, Messina, Italy; 6 Neuroradiology Unit, Biomedical Sciences and of Morphologic and Functional Images, University of Messina, Messina, Italy; 7 Faculty of Medicine and Surgery, University of Messina, Messina, Italy

**Keywords:** Stroke, Diffusion weighted imaging, Alberta stroke programme early computed tomography score, Fluid attenuated inversion recovery, Oxygen saturation, Activated partial thromboplastin time

## Abstract

**Background and Aim:**

The Diffusion-Weighted Imaging Alberta Stroke Programme Early Computed Tomography Score (DWI-ASPECTS) is a rapid and practical score used for quantifying the extent of early ischemic changes in acute ischemic stroke. It is strongly associated with stroke prognosis. The present study aimed to identify the main clinical, laboratory, and radiological features influencing DWI-ASPECTS in stroke patients with middle cerebral artery (MCA) occlusion.

**Methods:**

This retrospective study evaluated a cohort of patients with stroke due to MCA occlusion who underwent magnetic resonance imaging before thrombectomy. Clinical, laboratory, and imaging data were collected. Univariate and multivariable analyses were performed to assess associations with DWI-ASPECTS.

**Results:**

A total of 501 patients were selected. In the univariate analysis, a lower DWI-ASPECTS was associated with younger age, male sex, smoking, no atrial fibrillation, higher admission diastolic pressure, higher leukocyte count, lower activated partial thromboplastin time (aPTT), unknown onset time, FLAIR positivity, M1 occlusion, and internal carotid occlusion; a lower oxygen saturation was observed in patients with lower DWI-ASPECTS. Multivariable analysis confirmed the association of DWI-ASPECTS with age, sex, oxygen saturation, diastolic pressure, aPTT, unknown onset time, and FLAIR positivity.

**Conclusion:**

Our study shed light on several clinical, laboratory, and radiological factors influencing DWI-ASPECTS: age, sex, oxygen saturation, diastolic pressure, aPTT, unknown onset time, and FLAIR positivity. The present analysis was the first study to associate aPTT and oxygen saturation with DWI-ASPECTS. Both aPTT and oxygen saturation are modifiable factors that may represent potential targets to reduce ischemic damage and improve stroke prognosis.

**Supplementary Information:**

The online version contains supplementary material available at 10.1007/s10072-026-08903-x.

## Introduction

The Alberta Stroke Programme Early Computed Tomography Score (ASPECTS) is a widely used, rapid, and practical score for quantifying the extent of early ischemic changes in acute ischemic stroke [1, 2]. Several studies showed that ASPECTS is associated with stroke functional outcome [1, 3–5]. ASPECTS is also used to select patients for reperfusion therapies according to current guidelines from several scientific societies [6–8], and recent randomized controlled trials have demonstrated the benefits of mechanical thrombectomy (MT) in patients with low ASPECTS [9]. Compared to classical ASPECTS, the Diffusion-Weighted Imaging ASPECTS (DWI-ASPECTS) is more sensitive to early ischemic changes [10], and the modern imaging protocols are fast enough to allow the use of magnetic resonance imaging (MRI) in the acute phase. Furthermore, DWI-ASPECTS has a higher interrater agreement, and it is more accurate in predicting stroke outcome compared to classical ASPECTS [11]. Considering the clinical relevance of DWI-ASPECTS in guiding stroke management and treatment decisions, it is crucial to understand the underlying factors that can impact this score. The present study aimed to analyze the clinical, laboratory, and radiological features affecting DWI-ASPECTS in a cohort of stroke patients with large vessel occlusion.

## Methods

### Patients

In this retrospective analysis, we studied patients with acute ischemic stroke due to middle cerebral artery (MCA) occlusion who underwent MT. Data were retrieved from the prospective MT registry of a comprehensive stroke hub. The registry collected patients’ information from February 2014 to December 2020.

The inclusion criteria for the present study were:

- MCA occlusion.
- MRI protocol performed before MT.

The exclusion criteria were:

- Poor quality of MRI protocol that did not allow to evaluate DWI-ASPECTS.

### Clinical data

Baseline data and risk factors were assessed: age, sex, smoking, history of arterial hypertension, diabetes mellitus, previous stroke or transient ischemic attack (TIA), coronary artery disease, dyslipidemia, cancer history, and atrial fibrillation.

On admission, the following parameters were recorded: systolic blood pressure, diastolic blood pressure, and heart rate. Oxygen saturation was also recorded and measured using pulse oximetry.

We included the following laboratory tests on hospital admittance: glycemia, creatinine, white blood cell (WBC) count, platelets, prothrombin time (PT), activated partial thromboplastin time (aPTT), and international normalised ratio (INR). Low-density lipoprotein (LDL), high-density lipoprotein (HDL), triglycerides, and total cholesterol were also included, and they were dosed during the first day of hospitalization.

Stroke etiology was classified according to the Trial of ORG 10,172 in Acute Stroke Treatment criteria [12].

Admission neurological severity was evaluated using National Institutes of Health Stroke Scale (NIHSS).

Treatment with intravenous thrombolysis (IVT), onset-to-MRI time, and unknown onset were also recorded.

The good functional outcome at three months was also evaluated and was defined as a score ≤ 2 on the modified Rankin scale (mRs).

### Radiological data

Early ischemic changes were semi-quantitatively evaluated according to the DWI-ASPECTS [1, 10]. Fluid-Attenuated Inversion Recovery (FLAIR) positivity/negativity within the DWI lesion was recorded. Fazekas and Schmidt’s scale was applied to quantify leukoaraiosis [13]. Digital subtraction angiography (DSA) was used to determine the site of vessel occlusion. Carotid stenosis was assessed using color Doppler ultrasound and/or computed tomography (CT) angiography.

### Imaging features and analysis protocol

All MRI scans were performed using a 1.5 Tesla scanner (Ingenia, Philips Healthcare, Best, Netherlands). DWI characteristics were: TR/TE = 2946/86 ms; b-values = 0 and 1000 s/mm²; slice thickness = 5.0 mm with no interslice gap; voxel size = 1.5 × 2.2 × 5 mm; apparent diffusion coefficient (ADC) maps included; sequence duration = 60 s. FLAIR parameters were: TR/TE = 11,000/130 ms; TI = 2800 ms; slice thickness = 5.0 mm with no interslice gap; voxel size = 0.9 × 1.12 × 5 mm; sequence duration = 154 s. Two different biplane DSA systems were used: Artis BA and Artis Q Biplane (Siemens Healthcare GmbH, Germany). Two neuroradiologists (A.T. and E.M.) assessed the DWI-ASPECTS, parenchymal FLAIR positivity within DWI positive area, and leukoaraiosis grade. Two other raters (D.V. and M.L.) evaluated vessel occlusion site. Two workstations (DELL Precision 3630, CPU Xeon, 16GB RAM, Nvme SSD) were used, each equipped with identical high-resolution screens (BARCO thin film transistor liquid crystal display, 3MP, 20.8 inches, 2048 × 1536 resolution). The imaging software used was Suitestensa RIS (Ebit - Esaote Group). The modification of the image window and level was allowed in order to facilitate the radiological evaluation. A third observer (S.L.V.) was consulted to resolve any disagreements between raters. All radiologists had experience in stroke MRI and DSA reading and were blinded to clinical information.

### Study endpoints

The primary endpoint was to find the clinical, laboratory, and radiological features affecting the DWI-ASPECTS.

The secondary endpoints were to verify if admission neurological severity and 3-month functional outcome were also related to the same predictors of DWI-ASPECTS.

### Missing data

Variables with a missing data rate of 15% or higher were excluded from the study.

### Statistical analysis

#### Descriptive analyses and distribution

Categorical variables were expressed as absolute frequencies and percentages. Every continuous variable was reported as median with interquartile range (IQR). The Shapiro–Wilk test was used to evaluate the distribution normality.

#### Univariate analyses

The Mann–Whitney U test and Kruskal–Wallis test were performed for comparison between the categorical and continuous variables. Regarding the above-mentioned tests, mean ranks were provided in order to better show the significant differences between groups for DWI-ASPECTS analysis. Spearman’s rank correlation was used to assess the relationship between continuous variables. Chi-square test was used to evaluate the associations between categorical variables.

#### Multivariable analyses

Regarding DWI-ASPECTS, a model of ordinal logistic regression was built. It included all variables with *P*-value < 0.1 in the univariate analysis. In regard to admission NIHSS analyses, two models of multiple linear regression were built. A model included DWI-ASPECTS and other one excluded it. Furthermore, the models included all variables with *P*-value < 0.1 in the univariate analysis for admission NIHSS and significant variables from multivariable analysis of DWI-ASPECTS. Regarding the multivariable analyses for mRs ≤ 2, the multiple logistic regression was applied. A model included DWI-ASPECTS and other one excluded it. These models included all variables with *P*-value < 0.1 in the univariate analysis for mRs ≤ 2 and significant variables from multivariable analysis of DWI-ASPECTS. Collinearity among predictors was assessed using the Variance Inflation Factor (VIF) and the correlation matrix; critical collinearity was defined as VIF > 5 and/or *r* ≥ 0.8. In case of collinearity, separate models were developed, one for each collinear variable. Furthermore, a backward elimination method (threshold *P*-value > 0.1) was applied.

#### Significant threshold

A *P*-value ≤ 0.05 was considered significant.

#### Software

The statistical analyses were performed using R software.

## Results

### Study population and general characteristics

**Fig. 1:**
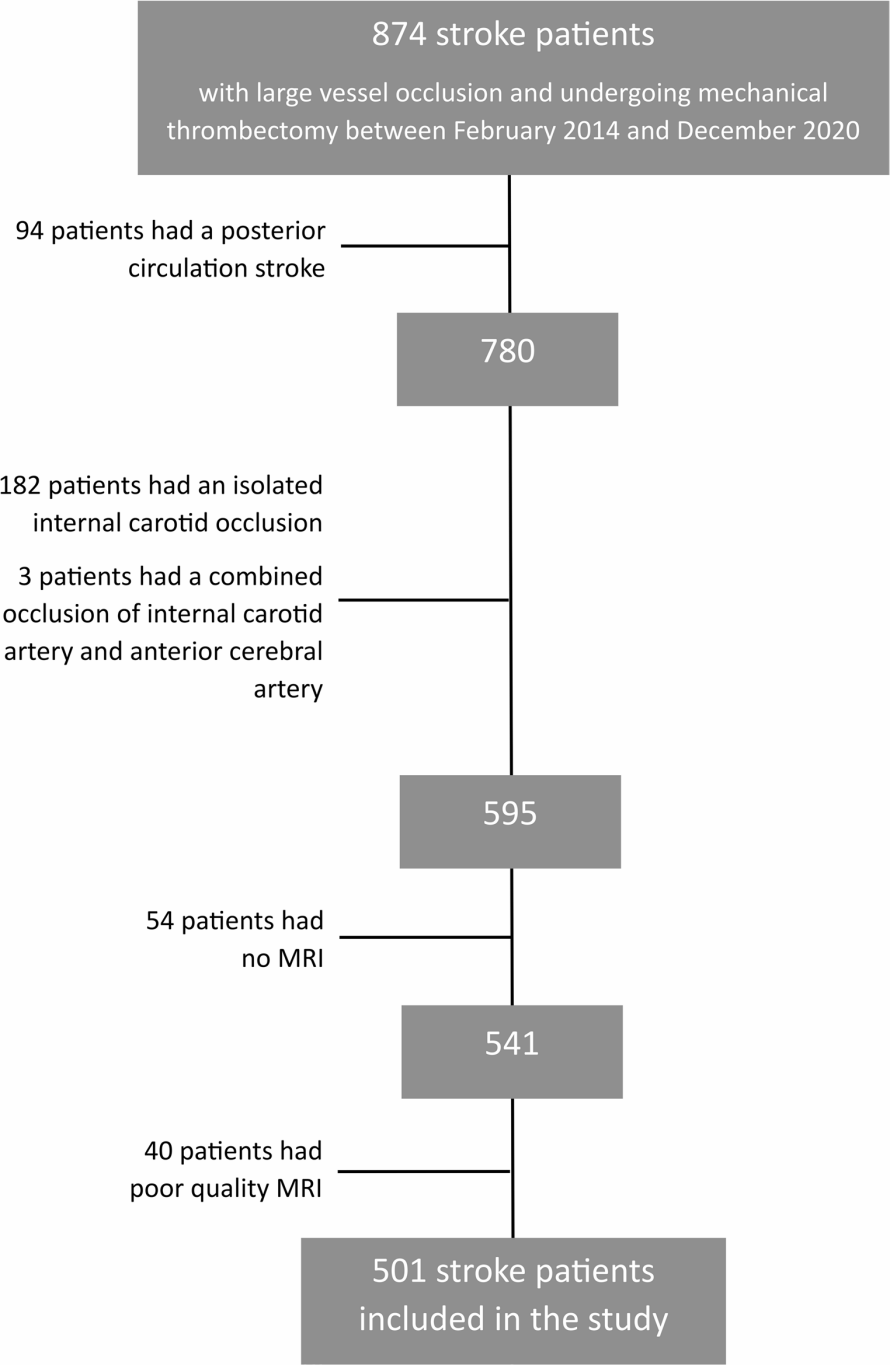
Included/excluded patients’ flow-chart

The present study selected 501 out of 874 consecutive stroke patients: 94 patients were excluded because of posterior circulation stroke; 182 had an isolated occlusion of internal carotid artery (ICA); 3 had combined occlusion of ICA and anterior cerebral artery; MRI was not performed in 54 cases (MRI was not available in 21 cases, 17 patients had a mechanical heart valve, 11 patients had a pacemaker or an implantable cardioverter-defibrillator, 3 had other non-MRI-compatible metallic prostheses, and 2 had metallic foreign bodies); and the MRI protocol was of poor quality in 40 patients. See Fig. 1.

The median age was 77 years. Two hundred thirteen patients (42.5%) were male. Atrial fibrillation was present in two hundred fifteen patients (44.3%). One hundred twenty-eight patients (29.6%) had a carotid stenosis ≥ 50%. Large artery atherosclerosis was the cause of stroke in eighty-five cases (17.4%), cardioembolism in one hundred eighty-nine (38.7%), and undetermined strokes were two hundred (41.0%). Four hundred two (80.2%) patients had an M1 occlusion and ninety-nine (19.8%) had an M2 occlusion. Furthermore, seventy-four patients (14.8%) presented also an ICA occlusion. The median DWI-ASPECTS was 7 (IQR 6–8). FLAIR was positive in three hundred sixty-six patients (79.0%). The median NIHSS on admission was 14 (IQR 10–18). IVT was performed in one hundred forty-four (29.0%) cases. A good functional outcome was achieved in one hundred sixty-nine (39.0%) patients.

The present and remaining data were summarized in Table 1. Details of missing data were provided in Supplementary Table S1.

**Table 1:** Demographics, baseline characteristics, and radiological features

	Overall
	*n* = 501
**Age** years, median (IQR)	77 (69–83)
**Male** n (%)	213 (42.5)
**Smoking** n (%)	85 (17.9)
**Arterial hypertension** n (%)	376 (77.5)
**Diabetes mellitus** n (%)	112 (23.1)
**Previous stroke/TIA** n (%)	74 (15.3)
**Coronary artery disease** n (%)	79 (16.3)
**Dyslipidemia** n (%)	150 (30.9)
**Cancer history** n (%)	57 (11.8)
**Atrial fibrillation** n (%)	215 (44.3)
**Admission systolic pressure** mmHg, median (IQR)	150 (135–167)
**Admission diastolic pressure** mmHg, median (IQR)	80 (70–90)
**Heart rate** bpm, median (IQR)	80 (71–88)
**Oxygen saturation** %, median (IQR)	97 (96–98)
**Admission blood glucose** mg/dL^†^, median (IQR)	128 (110–154)
**Creatinine** mg/dL^†^, median (IQR)	0.90 (0.80–1.20)
**WBC** 10^3^ cells/mm^3†^, median (IQR)	8.70 (7.12–10.78)
**Platelets** 10^3^ cells/mm^3†^, median (IQR)	222.00 (182.00-268.75)
**PT %**, median (IQR)	89.00 (79.00–97.00)
**aPTT** seconds^†^, median (IQR)	28.30 (26.20–31.20)
**INR**^†^, median (IQR)	1.07 (1.02–1.14)
**Total cholesterol** mg/dL^†^, median (IQR)	168.00 (141.00-193.25)
**LDL** mg/dL^†^, median (IQR)	92.00 (72.00-118.00)
**HDL** mg/dL^†^, median (IQR)	47.00 (39.00–57.00)
**Triglycerides** mg/dL^†^, median (IQR)	85.00 (67.00-110.00)
**Stroke etiology** n (%)	
Large artery atherosclerosis	85 (17.4)
Cardio embolism	189 (38.7)
Undetermined	200 (41.0)
Other causes	14 (2.9)
**Unknown onset time** n (%)	163 (33.5)
**Onset-MRI time** minutes, median (IQR)	202 (144–285)
**DWI-ASPECTS**, median (IQR)	7 (6–8)
**FLAIR positive** n (%)	366 (79.0)
**Fazekas scale**, median (IQR)	1 (1–2)
**Carotid stenosis ≥ 50%** n (%)	128 (29.6)
**ICA occlusion** n (%)	74 (14.8)
**MCA occlusion** n (%)	
M1	02 (80.2)
M2	99 (19.8)
**Admission NIHSS**, median (IQR)	14 (10–18)
**Intravenous thrombolysis** n (%)	144 (29.0)
**3-month mRs ≤ 2** n (%)	169 (39.0)

*IQR* interquartile range, *TIA* transitory ischemic attack, *WBC* white blood cells, *PT* prothrombin time, *aPTT* activated partial thromboplastin time, *INR* international normalized ratio, *LDL* low-density lipoprotein, *HDL* high-density lipoprotein, *MRI* magnetic resonance imaging, *DWI-ASPECTS* Diffusion-Weighted Imaging- Alberta stroke program early computed tomography score, *FLAIR* Fluid-Attenuated Inversion Recovery, *ICA* internal carotid artery, *MCA* middle cerebral artery, *NIHSS* National Institutes of Health Stroke Scale, *mRs* modified Rankin scale

**† Normal values**: Blood glucose (65–110); Creatinine (0.5–1.2); WBC (4.5-9.0); Platelets (150.0-350.0); PT (70–120); aPTT (21.0–35.0); INR (0.8–1.2); Total cholesterol (130–220); LDL (< 100); HDL (> 65); Triglycerides (50–60)

### DWI-ASPECTS analyses

#### Univariate analysis

Lower DWI-ASPECTS was observed in men (*P*-value = 0.00), in smokers (*P*-value = 0.05), and in patients without atrial fibrillation (*P*-value = 0.01). Furthermore, it was related to unknown onset time (*P*-value = 0.00). Lower DWI-ASPECTS was also associated with FLAIR positivity (*P*-value = 0.00), M1 occlusion (*P*-value = 0.01), and ICA occlusion (*P*-value = 0.01). Regarding the other qualitative variables, there were no significant associations. See Table 2, mean ranks were provided in order to better show the significant differences between groups.

**Table 2:** Univariate analysis for DWI-ASPECTS

Qualitative variables	Median (IQR)	Mean rank	*p*-value
Sex			
Male	7 (6–8)	227.65	0.00
Female	7 (6–8)	268.26	
Smoking			
Yes	7 (5–8)	212.23	0.05
No	7 (6–8)	243.62	
Arterial hypertension			
Yes	7 (6–8)	244.92	0.57
No	7 (6–8)	236.37	
Diabetes mellitus			
Yes	7 (6–8)	248.89	0.60
No	7 (6–8)	241.23	
Previous stroke/TIA			
Yes	7 (6–8)	236.92	0.70
No	7 (6–8)	243.51	
Coronary artery disease			
Yes	7 (6–8)	264.88	0.11
No	7 (6–8)	238.13	
Dyslipidemia			
Yes	7 (6–8)	251.76	0.35
No	7 (6–8)	239.08	
Cancer history			
Yes	7 (6–8)	235.99	0.72
No	7 (6–8)	242.80	
Atrial fibrillation			
Yes	7 (6–8)	261.22	0.01
No	7 (6–8)	228.49	
Stroke etiology			
Large artery atherosclerosis	7 (6–8)	224.84	0.34
Cardio embolism	7 (6–8)	256.14	
Undetermined	7 (6–8)	243.24	
Other causes	7 (6-7.75)	224.79	
Unknown onset			
Yes	7 (6–8)	215.42	0.00
No	7 (6–8)	257.67	
FLAIR			
Positive	7 (6–8)	222.33	0.00
Negative	7 (6–8)	268.51	
MCA occlusion site			
M1	7 (6–8)	242.36	0.01
M2	7 (6.5-8)	286.08	
ICA occlusion			
Yes	7 (5.25–7.75)	213.87	0.01
No	7 (6–8)	257.43	
Carotid stenosis			
≥ 50%	7 (6–8)	213.30	0.72
< 50%	7 (6–8)	217.84	
Quantitative variables	**Correlation coefficient**		***p*** **-value**
Age	0.171		0.00
Admission systolic pressure	-0.024		0.61
Admission diastolic pressure	-0.120		0.01
Heart rate	0.022		0.64
Oxygen saturation	0.090		0.06
Admission blood glucose	-0.021		0.66
Creatinine	0.045		0.33
Admission WBC	-0.110		0.02
Platelets	-0.054		0.25
PT	-0.026		0.58
aPTT	0.097		0.04
INR	0.043		0.37
Total cholesterol	0.006		0.90
LDL	-0.021		0.67
HDL	0.078		0.10*
Triglycerides	-0.069		0.15
Onset-MRI time	-0.074		0.19
Fazekas scale	-0.005		0.92

*IQR* interquartile range, *TIA* transitory ischemic attack, *FLAIR* Fluid-Attenuated Inversion Recovery, *MCA* middle cerebral artery, *ICA* internal carotid artery, *WBC* white blood cells, *PT* prothrombin time, *aPTT* activated partial thromboplastin time, *INR* international normalized ratio, *LDL* low-density lipoprotein, *HDL* high-density lipoprotein, *MRI* magnetic resonance imaging

*P-value rounded down and variable excluded from the multivariate analysis

DWI-ASPECTS was positively correlated with age (ρ = 0.17, *P*-value = 0.00) and aPTT (ρ = 0.10, *P*-value = 0.01). Negative correlations were found with admission diastolic pressure (ρ = -0.12, *P*-value = 0.01), and admission WBC (ρ = -0.11, *P*-value = 0.02). Regarding the other quantitative variables, there were no significant associations. A relevant trend was observed for oxygen saturation, but without a significant value on univariate analysis (ρ = 0.09, P- value = 0.06). See Table 2.

#### Multivariable analysis

Multivariable analysis confirmed that lower DWI-ASPECTS was independently associated with younger age (*P*-value = 0.00), male sex (*P*-value = 0.05), lower oxygen saturation (*P*-value = 0.05), higher admission diastolic pressure (*P*-value = 0.05), shorter aPTT (*P*-value = 0.02), unknown onset time (*P*-value = 0.01), and FLAIR positivity (*P*-value = 0.00). See Table 3.

**Table 3:** Multivariable analysis for DWI-ASPECTS

Multivariate analyses for DWI-ASPECTS		
	Initial Model	Final Model
	*p*-value	*p*-value
Age	0.01	0.00
Sex	0.12	0.05
Smoking	0.78	
Atrial fibrillation	0.70	
Oxygen saturation	0.00	0.05
Admission DP	0.02	0.05
Admission WBC	0.21	
aPTT	0.03	0.02
Unknown onset time	0.02	0.01
FLAIR	0.00	0.00
ICA occlusion	0.22	
MCA occlusion site	0.32	
*DP* diastolic pressure, *WBC* white blood cells, *aPTT* activated partial thromboplastin time, *FLAIR* Fluid-Attenuated Inversion Recovery, *ICA* internal carotid occlusion, *MCA* middle cerebral artery		

### Admission NIHSS analyses

#### Univariate analysis

Higher admission NIHSS was associated with previous stroke/TIA (*P*-value = 0.05) and M1 occlusion (*P*-value = 0.00). Regarding the other qualitative variables, there were no significant associations. Admission NIHSS was positively correlated with admission WBC (ρ = 0.12, *P*-value = 0.01) and Fazekas scale (ρ = 0.15, *P*-value = 0.00). Negative correlations were found with oxygen saturation (ρ = -0.14, *P*-value = 0.00), aPTT (ρ = -0.11, *P*-value = 0.02), and DWI-ASPECTS (ρ = -0.32, *P*-value = 0.00). Regarding the other quantitative variables, there were no significant associations. See Table S2.

#### Multivariable analyses

The model including DWI-ASPECTS showed that higher admission NIHSS was associated with older age (*P*-value = 0.03), higher admission WBC (*P*-value = 0.05), lower DWI-ASPECTS (*P*-value = 0.00), and M1 occlusion (*P*-value = 0.00). See Table S3.

The model excluding DWI-ASPECTS showed that higher admission NIHSS was related to higher admission WBC (*P*-value = 0.05), severe leukoaraiosis (*P*-value = 0.00), and M1 occlusion (*P*-value = 0.00). See Table S3.

### Good functional outcome analyses

#### Univariate analysis

A 3-month mRs 0–2 was associated with younger age (*P*-value = 0.00), smoking (*P*-value = 0.03), no medical history of arterial hypertension (*P*-value = 0.00), and no previous stroke/TIA (*P*-value = 0.01). Good functional outcome was also related to known onset time (*P*-value = 0.00) and no IVT (*P*-value = 0.02). Regarding the other qualitative variables, there were no significant associations. A relevant trend was observed for dyslipidemia, but without a significant value on univariate analysis (P- value = 0.06). See Table S4. Furthermore, a 3 month mRs 0–2 was associated with lower admission systolic blood pressure (*P*-value = 0.01), lower admission heart rate (*P*-value = 0.04), lower admission glycemia (*P*-value = 0.01), higher LDL levels (*P*-value = 0.04), higher DWI-ASPECTS (*P*-value = 0.00), lower score at Fazekas scale (*P*-value = 0.00), and lower admission NIHSS score (*P*-value = 0.00). Regarding the other quantitative variables, there were no significant associations. See Table S4.

#### Multivariable analyses

Collinearity was detected between total cholesterol and LDL. Four multivariable analysis models were developed: model A including DWI-ASPECTS and total cholesterol; model B including DWI-ASPECTS and LDL; model C including total cholesterol and excluding DWI-ASPECTS; and model D including LDL and excluding DWI-ASPECTS. See Table S5.

Model A showed that good functional outcome was associated with younger age (*P*-value = 0.00), no previous stroke/TIA (*P*-value = 0.03), dyslipidemia (*P*-value = 0.00), lower admission glycemia (*P*-value = 0.05), known onset time (*P*-value = 0.01), higher DWI-ASPECTS (*P*-value = 0.01), and lower admission NIHSS (*P*-value = 0.00).

In Model B, good functional outcome was related to younger age (*P*-value = 0.00), no previous stroke/TIA (*P*-value = 0.02), dyslipidemia (*P*-value = 0.01), known onset time (*P*-value = 0.01), higher DWI-ASPECTS (*P*-value = 0.00), and lower admission NIHSS (*P*-value = 0.00).

Model C and Model D showed that good functional outcome was associated with younger age (*P*-value = 0.00), no previous stroke/TIA (*P*-value = 0.01), dyslipidemia (*P*-value = 0.00), lower admission glycemia (*P*-value = 0.04), known onset time (*P*-value = 0.00), lower admission NIHSS (*P*-value = 0.00), and no treatment with IVT (*P*-value = 0.05).

## Discussion

The present study showed that DWI-ASPECTS was associated with several clinical, laboratory, and radiological features.

In our cohort, higher DWI-ASPECTS scores were observed in older patients, a finding consistent with previous studies [14–16]. This association could be explained through the reduced energy demand [17] and the chronic hypoperfusion of the elderly brain [18]. These elements may favour an increased ischemic tolerance and, consequently, a smaller infarct core [19]. Furthermore, in line with other literature data [14, 20], female patients presented higher DWI-ASPECTS comparing with male. This difference could be due to a better cerebrovascular autoregulation as illustrated by Deegan et al. [21]. In particular, several authors described a better collateral circulation in women [22–24]. It is interesting to observe that DWI-ASPECTS was not related with onset-to-MRI time in our study, but there was an association between this score and unknown onset time. It is plausible that patients with unknown onset time could have a much longer onset-to-MRI time than patients with known onset, and this may explain the lower DWI-ASPECTS, indirectly suggesting a possible role of time in the extension of DWI lesions in acute ischemic stroke. Moreover, previous studies reported an association between lesion size of ischemic stroke and unknown onset in literature [25, 26]. The present work showed also an association between higher diastolic blood pressure and lower DWI-ASPECTS, confirming the results of Inoue et al. study [27] and highlighting the role of high blood pressure on ischemic damage in the acute phase [28, 29].

Regarding radiological features, an association was found with FLAIR positivity, consistent with previous studies [30–32]. FLAIR is sensitive to vasogenic edema, and FLAIR hyperintensity could be the sign of advanced tissue changes that lead to an increase of the ischemic core. Moreover, this sequence is used as a tissue clock [30, 32], and it is noteworthy that we found an association with FLAIR positivity, but not with onset-to-MRI time in the multivariable analysis. This suggests that ischemic lesion extent may be more closely related to tissue changes than to time per se. From another point of view, it should be considered that FLAIR positivity may be more easily detected in a large lesion than in a small ischemic area, and this different difficulty may also explain the present association. One hypothesis does not necessarily exclude the other. It is remarkable to observe that the present study did not show an association between leukoaraiosis and DWI-ASPECTS. Literature data are controversial, some studies described an association between ASPECTS/stroke volume size and leukoaraiosis [20, 33, 34], whereas others did not confirm it [14, 15, 35]. Further studies are needed to clarify the influence of leukoaraiosis on the lesion size in acute ischemic stroke, given the importance of the present topic. The present research also showed that MCA occlusion site and ICA occlusion were related to DWI-ASPECTS on the univariate analysis, but the multivariable analysis did not show the association. Probably, MCA occlusion site and ICA occlusion are determining factors for the early ischemic changes, but there are other stronger predictors. Regarding literature data, some studies showed an association between the extension of early ischemic changes and the occlusion site [14, 34, 36, 37], whereas other studies did not confirm it [16, 27, 38, 39].

In regard to laboratory parameters, a shorter aPTT was related to a lower DWI-ASPECTS. The association between DWI-ASPECTS and aPTT was one of the most relevant findings in our study. Lin et al. showed that lower aPTT was related to higher stroke risk, more severe stroke, and neurological worsening [40]. Cho et al. found that lower aPTT was associated with higher admission NIHSS and larger DWI-lesion volumes in 46 stroke patients on dabigatran [41]. A lower aPTT value could be the marker of a procoagulant state that could increase the stroke risk and favour a larger ischemic lesion with more severe neurological deficits. Our study also demonstrated that lower DWI-ASPECTS was associated with reduced oxygen saturation levels. Local oxygen depletion is the basis of ischemic damage [42], and it is biologically plausible that a lower oxygen saturation could favour an increase of the core lesion size. Furthermore, recent studies found that oxygen therapy in stroke patients with large vessel occlusion could reduce the infarct volume measured at 24–48 h [43, 44]; our study revealed the effect of oxygen saturation on earliest ischemic changes. The present association was significant only after adjustments in our multivariable analyses, and further studies are needed to confirm it. From another point of view, a lower oxygen saturation may not be the cause, but rather the consequence of a larger ischemic lesion, potentially resulting in more severe neurological deficits and altered consciousness with respiratory involvement. These hypotheses are not mutually exclusive, and a bidirectional relationship may exist. Nevertheless, we found no association between oxygen saturation and neurological severity in the multivariable analysis in the present study. It is important to highlight that both aPTT and oxygen saturation are modifiable factors that may serve as potential therapeutic targets to reduce ischemic damage. Future research focused on optimizing these parameters during early acute phase may help to improve stroke prognosis.

In the present research, among DWI-ASPECTS predictors, only the age was a direct predictor of admission neurological severity, whereas only age and unknown onset time were associated with the functional outcome. Regarding the other DWI-ASPECTS predictors, they probably have an indirect role on admission neurological severity and functional outcome, influencing DWI-ASPECTS which was consistently associated with NIHSS and 3-month mRs.

A major strength of this study lies in its comprehensive analysis of several clinical, laboratory, and radiological features. In the literature, other studies focused only on radiological characteristics or only on laboratory data, our study was well balanced from this point of view and included a large cohort of patients. The present research was based on MRI that is more accurate to detect early ischemic changes compared to CT [10]. We studied DWI-ASPECTS which is easy and rapid to obtain in comparison to core volume in acute setting: it is used daily to evaluate stroke patients in several centers, and it is strongly associated with prognosis. It is important to highlight that DWI-ASPECTS and volume have not a univocal correspondence because every area is differently weighted in ASPECTS score, and a point is subtracted independently of lesion size in that specific region.

The present study had several limitations. The work had all the limitations inherent in retrospective studies. Significant associations did not imply a direct causal relationship. The study included patients from a single center; however, this choice allowed for data homogeneity. Collateral circulation was not analysed due to missing data in 34.2% of patients. We analysed a specific stroke population, and further studies are needed to validate the present findings in other stroke patients. In particular, patients were selected for MT treatment according to Italian and European guidelines in our study, and some patients could be excluded due to low ASPECTS and/or severe pre-stroke disability. Furthermore, the present results may not be totally generalizable to classical ASPECTS because of the differences between DWI and CT imaging. Finally, DWI may overestimate the ischemic core, indeed reversal of DWI-positive lesion was described in literature [45, 46]. A DWI-positive lesion likely includes regions with different perfusion status, and patients undergoing reperfusion therapies can sometimes obtain a reduction of early DWI-positive area [45, 46].

## Conclusion

DWI-ASPECTS has a relevant prognostic role in stroke patients, and understanding the factors that influence it is essential. The study shed light on several clinical, laboratory, and radiological factors influencing DWI-ASPECTS. To our knowledge, this was the first study to associate aPTT and oxygen saturation with DWI-ASPECTS. Our findings could be the basis of future research aimed at improving stroke prognosis and management: both aPTT and oxygen saturation are modifiable factors that could be potential targets in order to reduce ischemic damage.

## Supplementary Information

Below is the link to the electronic supplementary material.

Supplementary Material 1 (DOCX 22.6 KB)

Supplementary Material 2 (DOCX 29.8 KB)

Supplementary Material 3 (DOCX 24.0 KB)

Supplementary Material 4 (DOCX 29.2 KB)

Supplementary Material 5 (DOCX 29.5 KB)

## Data Availability

The data that support the findings of this study are available from the corresponding author upon reasonable request.

## Declarations

### Guidelines

The present study followed the Strengthening the reporting of observational studies in epidemiology (STROBE) guidelines.
