# Supplementary material for: Clinical, laboratory, and radiological features influencing admission DWI-ASPECTS in stroke patients with middle cerebral artery occlusion undergoing mechanical thrombectomy
Source: Neurol Sci. 2026 Mar 7;47(4):327. doi: 10.1007/s10072-026-08903-x (PMC12966226; doi:10.1007/s10072-026-08903-x)
Supplement: Supplementary file 1 — Supplementary Material 1 (DOCX 22.6 KB) [file 10072_2026_8903_MOESM1_ESM.docx]

**Table S1 Missing data**

|  | **N (%)** |
| --- | --- |
|  |  |
| **Age** | 0 (0.0) |
| **Sex** | 0 (0.0) |
| **Smoking** | 26 (5.2) |
| **Arterial hypertension** | 16 (3.2) |
| **Diabetes mellitus** | 16 (3.2) |
| **Previous stroke/TIA** | 17 (3.4) |
| **Dyslipidemia** | 16 (3.2) |
| **Cancer history** | 18 (3.6) |
| **Atrial fibrillation** | 16 (3.2) |
| **Admission systolic pressure** | 55 (11.0) |
| **Admission diastolic pressure** | 55 (11.0) |
| **Heart rate** | 69 (13.8) |
| **Oxygen saturation** | 67 (13.4) |
| **Admission blood glucose** | 59 (11.8) |
| **Creatinine** | 40 (8.0) |
| **WBC** | 40 (8.0) |
| **Platelets** | 39 (7.8) |
| **PT** | 57 (11.4) |
| **aPTT** | 56 (11.2) |
| **INR** | 50 (10.0) |
| **Total cholesterol** | 57 (11.4) |
| **LDL** | 59 (11.8) |
| **HDL** | 59 (11.8) |
| **Triglycerides** | 59 (11.8) |
| **Stroke etiology** | 13 (2.6) |
| **Unknown onset time** | 15 (3.0) |
| **Onset-MRI time** | 27 (8.0) |
| **DWI-ASPECTS** | 0 (0.0) |
| **FLAIR positive** | 38 (7.6) |
| **Fazekas scale** | 41 (8.2) |
| **Carotid stenosis ≥50%** | 69 (13.8) |
| **MCA occlusion** | 0 (0.0) |
| **ICA occlusion** | 0 (0.0) |
| **Admission NIHSS** | 7 (1.4) |
| **Intravenous thrombolysis** | 4 (0.8) |
| **3-month mRs 0-2** | 68 (13.6) |
| **TIA:** transitory ischemic attack; **WBC:** white blood cells; **PT**: prothrombin time; **aPTT**: activated partial thromboplastin time; **INR**: international normalized ratio; **LDL:** low-density lipoprotein; **HDL**: high-density lipoprotein; **MRI**: magnetic resonance imaging; **DWI-ASPECTS**: Diffusion-Weighted Imaging- Alberta stroke program early computed tomography score; **FLAIR**: Fluid-Attenuated Inversion Recovery; **MCA**: middle cerebral artery; **ICA**: internal carotid artery;  **NIHSS**: National Institutes of Health Stroke Scale;  **mRs**: modified Rankin scale. | |
