# Supplementary material for: Clinical, laboratory, and radiological features influencing admission DWI-ASPECTS in stroke patients with middle cerebral artery occlusion undergoing mechanical thrombectomy
Source: Neurol Sci. 2026 Mar 7;47(4):327. doi: 10.1007/s10072-026-08903-x (PMC12966226; doi:10.1007/s10072-026-08903-x)
Supplement: Supplementary file 2 — Supplementary Material 2 (DOCX 29.8 KB) [file 10072_2026_8903_MOESM2_ESM.docx]

**Table S2 Univariate analysis for admission NIHSS**

| **Qualitative variables** | **Median (IQR)** | **p-value** |
| --- | --- | --- |
| **Sex**  Male  Female | 14 (10-18)  14 (11-18) | 0.38 |
| **Smoking**  Yes  No | 14 (9-19)  14 (10-18) | 0.93 |
| **Arterial hypertension**  Yes  No | 14 (10-18)  13 (10-18) | 0.20 |
| **Diabetes mellitus**  Yes  No | 14 (10-17)  14 (10-18) | 0.38 |
| **Previous stroke/TIA**  Yes  No | 15 (12-18)  14 (10-18) | 0.05 |
| **Coronary artery disease**  Yes  No | 14 (10-19)  14 (10-18) | 0.99 |
| **Dyslipidemia**  Yes  No | 14 (10-18)  14 (10-18) | 0.62 |
| **Cancer history**  Yes  No | 15 (10-18)  14 (10-18) | 0.79 |
| **Atrial fibrillation**  Yes  No | 15 (10-18)  14 (10-18) | 0.19 |
| **Stroke etiology**  Large artery atherosclerosis  Cardio embolism  Undetermined  Other causes | 16 (10-18)  15 (10-18)  14 (10-17)  12 (10-16) | 0.26 |
| **Unknown onset**  Yes  No | 15 (11-19)  14 (10-18) | 0.11 |
| **FLAIR**  Positive  Negative | 14 (10-18)  14 (10-18) | 0.73 |
| **MCA occlusion site**  M1  M2 | 15 (11-18)  11 (6-16) | 0.00 |
| **ICA occlusion**  Yes  No | 16 (11-18)  14 (10-18) | 0.10* |
| **Carotid stenosis**  ≥50%  <50% | 13 (9-18)  14 (10-18) | 0.42 |
| **Quantitative variables** | **Correlation coefficient** | **p-value** |
| **Age** | 0.065 | 0.15 |
| **Admission systolic pressure** | 0.003 | 0.94 |
| **Admission diastolic pressure** | -0.026 | 0.59 |
| **Heart rate** | -0.067 | 0.16 |
| **Oxygen saturation** | -0.136 | 0.00 |
| **Admission blood glucose** | 0.076 | 0.11 |
| **Creatinine** | -0.029 | 0.53 |
| **Admission WBC** | 0.121 | 0.01 |
| **Platelets** | 0.073 | 0.12 |
| **PT** | -0.021 | 0.66 |
| **aPTT** | -0.114 | 0.02 |
| **INR** | 0.016 | 0.73 |
| **Total cholesterol** | -0.047 | 0.33 |
| **LDL** | -0.032 | 0.49 |
| **HDL** | 0.068 | 0.15 |
| **Triglycerides** | -0.029 | 0.55 |
| **Onset-MRI time** | -0.007 | 0.91 |
| **DWI-ASPECTS** | -0.318 | 0.00 |
| **Fazekas scale** | 0.146 | 0.00 |
| **IQR:** interquartile range; **TIA:** transitory ischemic attack; **FLAIR**: Fluid-Attenuated Inversion Recovery; **MCA**: middle cerebral artery; **ICA**: internal carotid artery; **WBC:** white blood cells; **PT**: prothrombin time; **aPTT**: activated partial thromboplastin time; **INR**: international normalized ratio; **LDL:** low-density lipoprotein; **HDL**: high-density lipoprotein; **MRI**: magnetic resonance imaging; **DWI-ASPECTS**: Diffusion-Weighted Imaging Alberta Stroke Programme Early Computed Tomography Score.  *P-value rounded-up and variable included in the multivariate analysis. | | |
