# Supplementary material for: Clinical, laboratory, and radiological features influencing admission DWI-ASPECTS in stroke patients with middle cerebral artery occlusion undergoing mechanical thrombectomy
Source: Neurol Sci. 2026 Mar 7;47(4):327. doi: 10.1007/s10072-026-08903-x (PMC12966226; doi:10.1007/s10072-026-08903-x)
Supplement: Supplementary file 3 — Supplementary Material 3 (DOCX 24.0 KB) [file 10072_2026_8903_MOESM3_ESM.docx]

**Table S3 Multivariable analysis for admission NIHSS**

| **Model including DWI-ASPECTS** | | |
| --- | --- | --- |
|  | **Initial**  **Model** | **Final**  **Model** |
|  | p-value | p-value |
| **Age** | 0.07 | 0.03 |
| **Sex** | 0.36 |  |
| **Previous stroke/TIA** | 0.32 |  |
| **Admission DP** | 0.09 |  |
| **Oxygen saturation** | 0.62 |  |
| **WBC** | 0.03 | 0.05 |
| **aPTT** | 0.19 |  |
| **Unknown onset time** | 0.52 |  |
| **DWI-ASPECTS** | 0.00 | 0.00 |
| **FLAIR positive** | 0.31 |  |
| **Fazekas scale** | 0.06 | 0.07 |
| **MCA occlusion** | 0.00 | 0.00 |
| **ICA occlusion** | 0.40 |  |
| **Model excluding DWI-ASPECTS** | | |
|  | **Initial**  **Model** | **Final**  **Model** |
|  | p-value | p-value |
| **Age** | 0.53 |  |
| **Sex** | 0.72 |  |
| **Previous stroke/TIA** | 0.34 |  |
| **Admission DP** | 0.36 |  |
| **Oxygen saturation** | 0.32 |  |
| **WBC** | 0.03 | 0.00 |
| **aPTT** | 0.06 |  |
| **Unknown onset time** | 0.19 |  |
| **FLAIR positive** | 0.98 |  |
| **Fazekas scale** | 0.01 | 0.00 |
| **MCA occlusion** | 0.00 | 0.00 |
| **ICA occlusion** | 0.19 |  |
| **TIA:** transitory ischemic attack **;DP**: diastolic pressure; **WBC**: white blood cells; **aPTT**: activated partial thromboplastin time; **DWI-ASPECTS**: Diffusion-Weighted Imaging Alberta Stroke Programme Early Computed Tomography Score; **FLAIR**: Fluid-Attenuated Inversion Recovery; **MCA**: middle cerebral artery, **ICA**: internal carotid artery. | | |
