# Supplementary material for: Clinical, laboratory, and radiological features influencing admission DWI-ASPECTS in stroke patients with middle cerebral artery occlusion undergoing mechanical thrombectomy
Source: Neurol Sci. 2026 Mar 7;47(4):327. doi: 10.1007/s10072-026-08903-x (PMC12966226; doi:10.1007/s10072-026-08903-x)
Supplement: Supplementary file 4 — Supplementary Material 4 (DOCX 29.2 KB) [file 10072_2026_8903_MOESM4_ESM.docx]

**Table S4 Univariate analysis for 3-month mRs ≤ 2**

|  | **mRs ≤ 2** | **mRs > 2** | **P-value** |
| --- | --- | --- | --- |
| **Age** years, median (IQR) | 73 (63-81) | 79 (72-84) | 0.00 |
| **Sex** n (%)  Male  Female | 83 (49.1)  86 (50.9) | 104 (39.4)  160 (60.6) | 0.06 |
| **Smoking** n (%)  Yes  No | 40 (24.0)  127 (76.0) | 38 (15.1)  214 (84.9) | 0.03 |
| **Arterial hypertension** n (%)  Yes  No | 116 (69.0)  52 (31.0) | 214 (83.3)  43 (16.7) | 0.00 |
| **Diabetes mellitus** n (%)  Yes  No | 36 (21.4)  132 (78.6) | 66 (25.7)  191 (74.3) | 0.37 |
| **Previous stroke/TIA** n (%)  Yes  No | 17 (10.1)  151 (89.9) | 51 (19.8)  206 (80.2) | 0.01 |
| **Coronary artery disease** n (%)  Yes  No | 28 (16.7)  140 (83.3) | 42 (16.3)  215 (83.7) | 1 |
| **Dyslipidemia** n (%)  Yes  No | 61 (36.3)  107 (63.7) | 70 (27.2)  187 (72.8) | 0.06 |
| **Cancer history** n (%)  Yes  No | 17 (10.1)  151 (89.9) | 30 (11.7)  226 (88.3) | 0.72 |
| **Atrial fibrillation** n (%)  Yes  No | 69 (41.1)  99 (58.9) | 125 (48.6)  132 (51.4) | 0.15 |
| **Admission systolic pressure** mmHg, median (IQR) | 148 (132-161) | 150 (137-170) | 0.01 |
| **Admission diastolic pressure** mmHg, median (IQR) | 80 (70-90) | 80 (70-90) | 0.60 |
| **Heart rate** bpm, median (IQR) | 78 (70-86) | 80 (72-90) | 0.04 |
| **Oxygen saturation** %, median (IQR) | 97 (96-98) | 97 (96-98) | 0.15 |
| **Admission blood glucose** mg/dL^†^, median (IQR) | 125 (106-146) | 132 (113-163) | 0.01 |
| **Creatinine** mg/dL ^†^, median (IQR) | 0.9 (0.8-1.2) | 0.9 (0.8-1.2) | 0.43 |
| **WBC** 10^3^ cells/mm^3†^, median (IQR) | 8.70 (7.13-10.62) | 9.00 (7.25-11.02) | 0.35 |
| **Platelets** 10^3^ cells/mm^3†^, median (IQR) | 225.00 (194.50-272.00) | 222.00 (188.00-268.00) | 0.42 |
| **PT %**, median (IQR) | 90 (82-97) | 89 (79-97) | 0.50 |
| **aPTT** seconds^†^, median (IQR) | 28.30 (26.15-30.75) | 28.00 (26.05-31.20) | 0.98 |
| **INR**^†^, median (IQR) | 1.06 (1.02-1.11) | 1.07 (1.02-1.15) | 0.31 |
| **Total cholesterol** mg/dL^†^, median (IQR) | 172 (145-195) | 163 (138-191) | 0.07 |
| **LDL** mg/dL^†^, median (IQR) | 94 (76-120) | 87 (67-116) | 0.04 |
| **HDL** mg/dL^†^, median (IQR) | 45 (38-55) | 48 (40-58) | 0.13 |
| **Triglycerides** mg/dL^†^, median (IQR) | 89 (68-110) | 84 (64-112) | 0.20 |
| **Stroke etiology** n (%)  Large artery atherosclerosis  Cardio embolism  Undetermined  Other causes | 24 (14.3)  68 (40.5)  68 (40.5)  8 (4.8) | 53 (20.6)  105 (40.9)  95 (37.0)  4 (1.6) | 0.10* |
| **Unknown onset time** n (%)  Yes  No | 41 (24.7)  125 (75.3) | 104 (40.0)  156 (60.0) | 0.00 |
| **Onset-MRI time** minutes, median (IQR) | 181 (140-261) | 199 (144-286) | 0.25 |
| **DWI-ASPECTS**, median (IQR) | 8 (7-8) | 7 (6-8) | 0.00 |
| **FLAIR positive** n (%)  Yes  No | 118 (77.1)  35 (22.9) | 196 (79.4)  51 (20.6) | 0.69 |
| **Fazekas scale,** median (IQR) | 1 (1-2) | 2 (1-2) | 0.00 |
| **Carotid stenosis ≥50%** n (%) | 42 (25.9)  120 (74.1) | 74 (33.5)  147 (66.5) | 0.14 |
| **ICA occlusion** n (%)  Yes  No | 18 (10.7)  151 (89.3) | 44 (16.7)  220 (83.3) | 0.11 |
| **MCA occlusion site** n (%)  M1  M2 | 130 (76.9)  39 (23.1) | 214 (81.1)  50 (18.9) | 0.36 |
| **Admission NIHSS,** median (IQR) | 11 (7-15) | 16 (12-19) | 0.00 |
| **Intravenous thrombolysis** n (%)  Yes  No | 40 (24.0)  127 (76.0) | 93 (35.2)  171 (64.8) | 0.02 |
| **mRs**: modified Rankin scale; **IQR:** interquartile range; **TIA:** transitory ischemic attack; **WBC:** white blood cells; **NLR**: neutrophil-to-lymphocyte ratio; **PT**: prothrombin time; **aPTT**: activated partial thromboplastin time; **INR**: international normalized ratio; **LDL:** low-density lipoprotein; **HDL**: high-density lipoprotein; **MRI**: magnetic resonance imaging; **DWI-ASPECTS**: Diffusion-Weighted Imaging- Alberta stroke program early computed tomography score; **FLAIR**: Fluid-Attenuated Inversion Recovery; **ICA**: internal carotid artery; **MCA**: middle cerebral artery; **NIHSS**: National Institutes of Health Stroke Scale.  **†** **Normal values**: Blood glucose (65-110); Creatinine (0.5-1.2); WBC (4.5-9.0); Platelets (150.0-350.0); PT (70-120); aPTT (21.0-35.0); INR (0.8-1.2); Total cholesterol (130-220); LDL (<100); HDL (>65); Triglycerides (50-60).  *P-value rounded-up and variable included in the multivariate analysis. | | | |
