# Supplementary material for: Clinical, laboratory, and radiological features influencing admission DWI-ASPECTS in stroke patients with middle cerebral artery occlusion undergoing mechanical thrombectomy
Source: Neurol Sci. 2026 Mar 7;47(4):327. doi: 10.1007/s10072-026-08903-x (PMC12966226; doi:10.1007/s10072-026-08903-x)
Supplement: Supplementary file 5 — Supplementary Material 5 (DOCX 29.5 KB) [file 10072_2026_8903_MOESM5_ESM.docx]

**Table S5 Multivariable analysis for 3-month mRs ≤ 2**

| **Model A** | | | **Model B** | | |
| --- | --- | --- | --- | --- | --- |
|  | **Initial**  **Model** | **Final**  **Model** |  | **Initial**  **Model** | **Final**  **Model** |
|  | p-value | p-value |  | p-value | p-value |
| **Age** | 0.00 | 0.00 | **Age** | 0.00 | 0.00 |
| **Sex** | 0.47 |  | **Sex** | 0.48 |  |
| **Smoking** | 0.99 |  | **Smoking** | 0.99 |  |
| **Arterial hypertension** | 0.41 | 0.06 | **Arterial hypertension** | 0.43 |  |
| **Previous stroke/TIA** | 0.01 | 0.03 | **Previous stroke/TIA** | 0.01 | 0.02 |
| **Dyslipidemia** | 0.02 | 0.00 | **Dyslipidemia** | 0.03 | 0.01 |
| **Admission SP** | 0.44 |  | **Admission SP** | 0.46 |  |
| **Admission DP** | 0.96 |  | **Admission DP** | 0.96 |  |
| **Heart rate** | 0.49 |  | **Heart rate** | 0.50 |  |
| **Oxygen saturation** | 0.41 |  | **Oxygen saturation** | 0.49 |  |
| **Admission glycemia** | 0.15 | 0.05 | **Admission glycemia** | 0.15 |  |
| **aPTT** | 0.79 |  | **aPTT** | 0.75 |  |
| **Total cholesterol** | 0.41 |  | **LDL** | 0.38 |  |
| **Stroke etiology** | 0.89 |  | **Stroke etiology** | 0.86 |  |
| **Unknown onset time** | 0.02 | 0.01 | **Unknown onset time** | 0.02 | 0.01 |
| **DWI-ASPECTS** | 0.02 | 0.01 | **DWI-ASPECTS** | 0.02 | 0.00 |
| **FLAIR positive** | 0.25 |  | **FLAIR positive** | 0.22 |  |
| **Fazekas scale** | 0.35 |  | **Fazekas scale** | 0.38 |  |
| **Admission NIHSS** | 0.00 | 0.00 | **Admission NIHSS** | 0.00 | 0.00 |
| **IVT** | 0.35 | 0.07 | **IVT** | 0.39 | 0.07 |
| **Model C** | | | **Model D** | | |
|  | **Initial**  **Model** | **Final**  **Model** |  | **Initial**  **Model** | **Final**  **Model** |
|  | p-value | p-value |  | p-value | p-value |
| **Age** | 0.01 | 0.00 | **Age** | 0.01 | 0.00 |
| **Sex** | 0.52 |  | **Sex** | 0.55 |  |
| **Smoking** | 0.99 |  | **Smoking** | 0.99 |  |
| **Arterial hypertension** | 0.39 |  | **Arterial hypertension** | 0.40 |  |
| **Previous stroke/TIA** | 0.01 | 0.01 | **Previous stroke/TIA** | 0.01 | 0.01 |
| **Dyslipidemia** | 0.02 | 0.00 | **Dyslipidemia** | 0.02 | 0.00 |
| **Admission SP** | 0.49 |  | **Admission SP** | 0.52 |  |
| **Admission DP** | 0.69 |  | **Admission DP** | 0.68 |  |
| **Heart rate** | 0.66 |  | **Heart rate** | 0.66 |  |
| **Oxygen saturation** | 0.31 |  | **Oxygen saturation** | 0.38 |  |
| **Admission glycemia** | 0.15 | 0.04 | **Admission glycemia** | 0.15 | 0.04 |
| **aPTT** | 0.89 |  | **aPTT** | 0.83 |  |
| **Total cholesterol** | 0.28 |  | **LDL** | 0.28 |  |
| **Stroke etiology** | 0.85 |  | **Stroke etiology** | 0.92 |  |
| **Unknown onset time** | 0.01 | 0.00 | **Unknown onset time** | 0.01 | 0.00 |
| **FLAIR positive** | 0.36 |  | **FLAIR positive** | 0.32 |  |
| **Fazekas scale** | 0.43 |  | **Fazekas scale** | 0.45 |  |
| **Admission NIHSS** | 0.00 | 0.00 | **Admission NIHSS** | 0.00 | 0.00 |
| **IVT** | 0.27 | 0.05 | **IVT** | 0.32 | 0.05 |
| **TIA:** transient ischemic attack; **SP**: systolic pressure; **DP**: diastolic pressure; **aPTT**: activated partial thromboplastin time; **LDL**: low-density lipoprotein; **DWI-ASPECTS**: Diffusion-Weighted Imaging Alberta Stroke Programme Early Computed Tomography Score; **FLAIR**: Fluid-Attenuated Inversion Recovery; **NIHSS**: National Institutes of Health Stroke Scale; **IVT**: intravenous thrombolysis.  **Model A** included total cholesterol and DWI-ASPECTS; **Model B** included LDL and DWI-ASPECTS; **Model C** included total cholesterol and excluded DWI-ASPECTS; **Model D** included LDL and excluded DWI-ASPECTS. | | | | | |
